# Outcomes and Predictors of Failure of Ultrasound Cyclo Plasty for Primary Open-Angle Glaucoma

**DOI:** 10.3390/jcm11226770

**Published:** 2022-11-16

**Authors:** Faisal A. Almobarak, Ahmed Alrubean, Waleed Alsarhani, Abdullah Aljenaidel, Essam A. Osman

**Affiliations:** 1 Department of Ophthalmology, College of Medicine, King Saud University, Riyadh 11451, Saudi Arabia; 2 Glaucoma Research Chair, King Saud University, Riyadh 11451, Saudi Arabia; 3 Department of Ophthalmology, College of Medicine, Al-Imam Muhammad Ibn Saud Islamic University, Riyadh 11564, Saudi Arabia; 4 Department of Ophthalmology, King Faisal Specialist Hospital and Research Center, Riyadh 11564, Saudi Arabia; 5 Department of Ophthalmology and Vision Sciences, University of Toronto, Toronto, ON M5T 3A9, Canada

**Keywords:** glaucoma, ciliary body, ultrasound cyclo plasty, primary open-angle glaucoma

## Abstract

**Aims:** To evaluate the outcomes of ultrasound cyclo plasty (UCP) for primary open-angle glaucoma (POAG) and identify the predictors of failure. **Methods:** This retrospective cohort study included patients with POAG who underwent UCP at King Abdul Aziz University Hospital, Riyadh, Saudi Arabia, between 2016 and 2021. The main outcome measures were the intraocular pressure (IOP), the number of antiglaucoma medications, and the presence of vision-threatening complications. The surgical outcome of each eye was based on the main outcome measures. Cox proportional hazard regression analysis was performed to identify the possible predictors of UCP failure. **Results:** Sixty-six eyes of fifty-five patients were included herein. The mean follow-up period was 28.95 (±16.9) months. The mean IOP decreased significantly from 23.02 (±6.1) to 18.22 (±7.0) and 16.44 (±5.3) mm Hg on the 12th and 24th months, respectively; the mean number of antiglaucoma medications decreased significantly from 3.23 (±0.9) to 2.15 (±1.5) and 2.09 (±1.6), respectively. The cumulative probabilities of overall success were 71.2 ± 5.6% and 40.9 ± 6.1% on the 12th and 24th months, respectively. High baseline IOP and the number of antiglaucoma medications were associated with a higher risk of failure (hazard ratio = 1.10 and 3.01, *p* = 0.04 and *p* < 0.01, respectively). The most common complications were cataract development or progression (30.8%) and prolonged or rebound anterior chamber reaction (10.6%). **Conclusions:** UCP reasonably controls the IOP and reduces the antiglaucoma medication burden in eyes with POAG. Nevertheless, the success rate is modest, with a high baseline IOP and number of medications.

## 1. Introduction

Glaucoma is a group of ocular disorders characterised by progressive damage of retinal ganglion cells and the optic nerve. It is the leading cause of irreversible blindness, affecting nearly 80 million people worldwide, and this number is expected to reach 111.8 million by 2040 [1]. The significant morbidity of glaucoma presents remarkable health, societal, and economic burden [2]. Primary open-angle glaucoma (POAG) is the most common type of glaucoma, accounting for 60–90% of glaucoma cases across different ethnic groups but far less in the Saudi population (12.8%) [1,3,4]. Several studies have demonstrated that lowering the intraocular pressure (IOP) is the principal factor for reducing the glaucoma progression rate and for preserving sight. The traditional treatment algorithm aims to achieve a balance between aqueous humour inflow and outflow by decreasing production and/or increasing drainage and, therefore, IOP control. When topical medications or laser therapy do not achieve adequate IOP control, incisional surgery should be considered. However, the postoperative complications should be taken into consideration before proceeding with such an option. Another option to reduce aqueous humour production is by destroying the ciliary body using different physical methods, such as laser therapy and cryotherapy [5,6]. However, the non-selective action of such cyclodestructive procedures over the target tissues and the arbitrary dose–effect relationship have resulted in significant risks of chronic hypotony, phthisis, uveal inflammation, macular oedema, and retinal detachment [7,8,9].

Ultrasound cyclo plasty (UCP) has been developed to achieve selective and controlled thermal effects on the ciliary body using high-intensity focused ultrasound. This procedure enables good tissue targeting and precise temperature control, resulting in the remodelling of the ciliary body by removing the epithelium and preserving the blood–aqueous barrier [10]. Both the miniaturised transducers and the circular-shaped probe matching the three-dimensional anatomy of the ciliary body allow correct focusing on the target tissue. Several studies reported encouraging results regarding the safety and efficacy of UCP [11,12,13,14,15,16,17]. Nevertheless, reports on the outcome of such a procedure in a major glaucoma type, such as POAG, which is a leading cause of blindness worldwide, are limited. We have previously described the outcomes of UCP in patients with different glaucoma types [16]. Since the literature lacks studies focusing on the outcomes of UCP in a major glaucoma type such as POAG, we were interested in evaluating the outcomes of UCP for uncontrolled POAG.

## 2. Materials and Methods

This retrospective study was approved by the institutional review board of King Saud University (E-22-6738), which is a part of a larger study on the outcomes of UCP, and all procedures adhered to the tenets of the Declaration of Helsinki. We reviewed the medical records of patients with POAG who underwent UCP between May 2016 and May 2021 at King Abdul Aziz University Hospital, Riyadh, Saudi Arabia. The inclusion criteria were as follows: medically uncontrolled IOP of ≥ 21 mm Hg despite maximum tolerated antiglaucoma medications; glaucomatous optic nerve head damage and open angle on gonioscopy; and a minimum follow-up period of 6 months. Meanwhile, the exclusion criteria were as follows: normal-tension glaucoma; secondary open-angle glaucoma; pregnancy; use of systemic medications that could affect the IOP; history of either refractive surgery, retinal detachment, or ocular tumour; and ocular infection 2 weeks prior to UCP. We followed the previous methods described by Almobarak et al. [16].

### 2.1. Surgical Methods

All procedures were performed using the EyeOP1 device (Eye Tech Care, Rillieux-la-Pape, France). The device consists of a single-use sterile pack, including a coupling cone and treatment probe of three sizes based on the biometric eye readings to best adapt to the eye, and a compact operator console. The surgeon’s name and the patient’s demographic data were registered, followed by the connection of the selected probe to the console. Thereafter, the machine automatically detected the probe, and the suction test began after clumping the suction probe. One of several staff glaucoma specialists credentialed for the procedure performed the surgery under peribulbar anaesthesia. The coupling cone was placed and adjusted on the centre of the patient’s eye by visualising an equal white scleral ring surrounding the cornea. The coupling cone was kept in place via vacuum suction activated using a foot pedal and was then filled with a balanced salt solution to allow ultrasound transmission. A second-generation probe, consisting of six piezoelectric transducers, was used for all patients. The transducers were automatically activated at a frequency of 21 MHz and an acoustic power of 2.45 W, with an 8-s duration for each sector and a 20-s pause between each treatment to allow complete evacuation of heat. The UCP procedures were performed by authors FA, EO, and SA. Postoperatively, all patients were treated with topical prednisolone drops four times a day for 1 week, with the frequency tapered gradually on a weekly basis. Antiglaucoma drops were resumed postoperatively based on the surgeon’s preference according to the case severity and IOP. The IOP was measured using the Goldmann applanation tonometer. The postoperative visits considered for this study were those conducted on the first postoperative day and at 2 to 4 weeks, 3 months, 6 months, 9 months, 12 months, 18 months, and 24 months.

### 2.2. Data Analysis

Pre- and postoperative data were collected for the following variables: age, sex, IOP, number of antiglaucoma medications, best-corrected visual acuity converted into logarithm of minimal angle of resolution (logMAR) format, time to failure, postoperative complications, and the need for subsequent pressure-lowering procedures to control the IOP. The variables were evaluated using Student’s *t*-test and the Wilcoxon rank test. Kaplan–Meier life table analysis was performed to estimate the success rate over the postoperative period and is presented as percentages ± standard errors. Success was classified as follows: (i) overall success (IOP reduction of ≥20% from the baseline level and IOP between 6 and 21 mm Hg with or without antiglaucoma medications); (ii) complete success (IOP reduction of ≥20% from the baseline level and IOP between 6 and 21 mm Hg without antiglaucoma medications); (iii) qualified success (the same as absolute success but with antiglaucoma medications); and (iv) failure (when any of the following develops: IOP reduction of <20% from the baseline level and IOP of >21 mm Hg despite maximum tolerated antiglaucoma medications on two visits, persistent hypotony (IOP of ≤5 mm Hg) on two visits causing hypotony maculopathy, the need for a higher number of glaucoma medications compared with the preoperative baseline, loss of vision due to glaucoma progression, postoperative vision-threatening complications, or the need for further glaucoma procedures to control the IOP, including a repeat UCP). IOP spikes were considered when the IOP was more than 30 mm Hg or increased by >10 mm Hg compared with the preoperative baseline. Hazards ratios (HRs) and confidence intervals (CIs) were calculated using the Cox proportional hazard regression analysis to evaluate the impact of the baseline characteristics on survival. The variables were presented as means and standard deviations, and *p*-values of <0.05 were considered statistically significant. Statistical analyses were performed using SPSS version 23 (IBM Corp., Armonk, NY, USA).

## 3. Results

**Figure 1:**
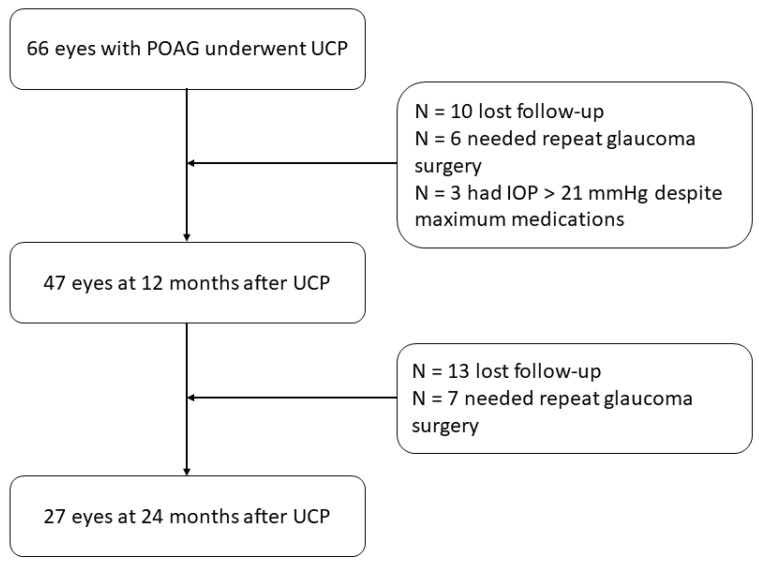
Flow chart of the study. UCP: Ultrasound cyclo plasty.

The study included 66 eyes of 55 patients. A flow chart is displayed in Figure 1. Twenty-nine eyes received previous glaucoma surgery, while 31 eyes received previous non-glaucoma surgery, mostly cataract surgery. The mean follow-up period was 28.95 (±16.9) months. Thirty-nine eyes were pseudophakic, while the remaining eyes were phakic and aphakic (Table 1). Among the 26 phakic eyes, 21 showed cataractous changes before surgery.

**Figure 2:**
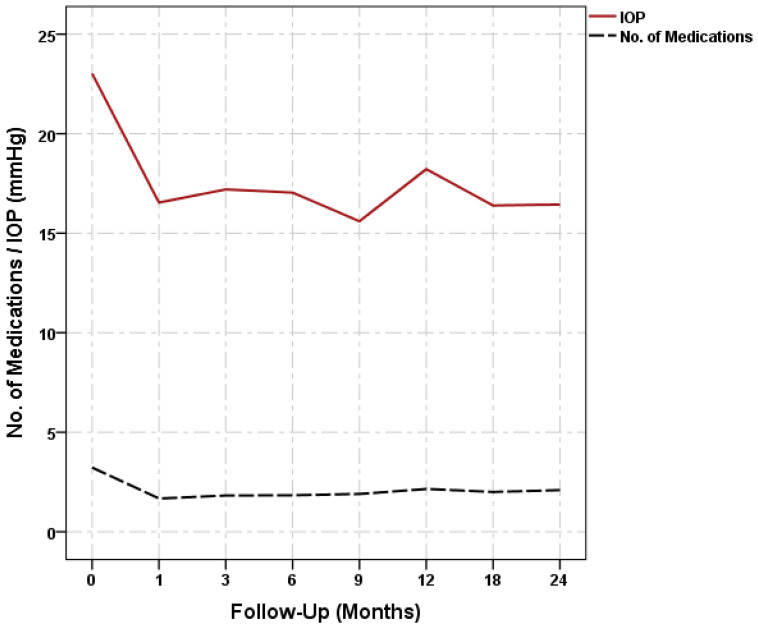
Changes in IOP and antiglaucoma medications.

The mean IOP decreased significantly from 23.02 (±6.1) to 18.22 (±7.0), 16.39 (±4.4), and 16.44 (±5.3) mm Hg on the 12th, 18th, and 24th months, respectively; the mean number of antiglaucoma medications decreased significantly from 3.23 (±0.9) to 2.15 (±1.5), 2.00 (±1.6), and 2.09 (±1.6), respectively; both parameters decreased throughout the follow-up period (*p* < 0.01) (Figure 2). There was a significant change in the mean logMAR during the first day and the third month postoperatively (Table 2). The most common postoperative complications were cataract development or progression in 8 eyes out of 26 phakic eyes (30.8%), of which 6 eyes (23.1%) required cataract surgery; prolonged or rebound anterior chamber reaction in 7 eyes (10.6%); hypotony with choroidal detachment in 2 eyes (3.0%); and macular oedema in 2 eyes (3.0%) (Table 3).

**Figure 3:**
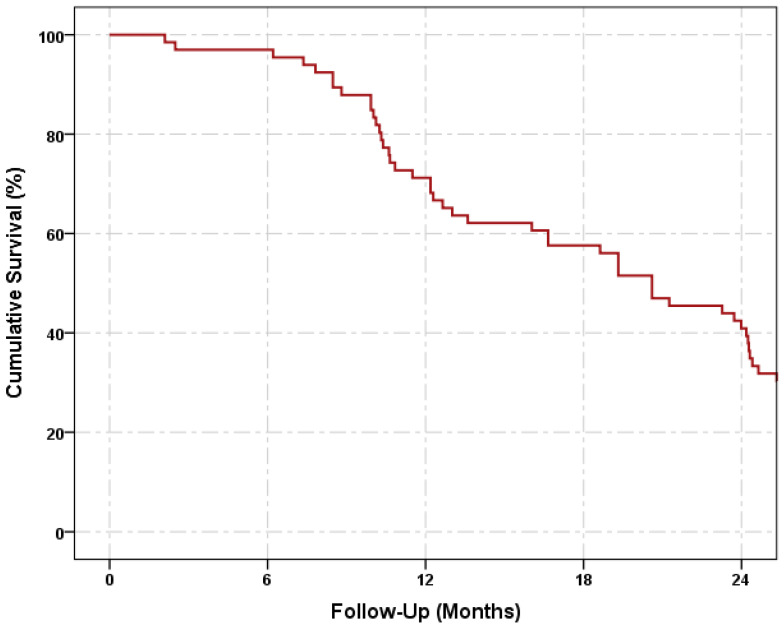
Kaplan–Meier survival curve showing the cumulative probability of success.

The cumulative probabilities of overall success were 71.2 ± 5.6%, 57.6 ± 6.1%, and 40.9 ± 6.1% on the 12th, 18th, and 24th months, respectively (Figure 3). The complete success rates were 78.6 ± 11.0%, 64.3 ± 12.8%, and 57.1 ± 13.2%, while the qualified success rates were 79.4 ± 6.9%, 67.6 ± 8.0%, and 50.0 ± 8.6% on the 12th, 18th, and 24th months, respectively. The Cox proportional hazard analyses of survival showed that the baseline IOP (HR = 1.10, 95% CI = 1.00–1.12, *p* = 0.04) and number of antiglaucoma medications (HR = 3.01, 95% CI = 1.61–5.60, *p* < 0.01) were both significant risk factors for failure. Age, sex, axial length, white-to-white diameter, history of old glaucoma surgery, and history of old non-glaucoma surgery were not significant risk factors for failure (Table 4).

In total, 16 eyes (24.2%) failed because of the following: 13 eyes (19.7%) had an uncontrolled IOP requiring further glaucoma surgery (3 eyes required a second glaucoma surgery to control the IOP), and 3 eyes (4.5%) had glaucoma progression with an uncontrolled IOP (1 eye had complete loss of vision) (Table 5). The mean time to failure was 11.73 (±6.2; range = 2.10–23.98) months. Most failures occurred 6 months after UCP.

## 4. Discussion

The current study showed that UCP was safe and effective for significantly reducing the IOP in patients with POAG. There were significant differences in the IOP and antiglaucoma medication burden on the 6th, 12th, 18th, and 24th months after a single UCP. The rate of IOP reduction ranged between 28.8% and 42.0% throughout the follow-up period, and the IOP was <18 mm Hg. Although the number of antiglaucoma medications decreased significantly after UCP, a gradual increment was needed to maintain a controlled IOP. We have previously described the outcomes of UCP in patients with different glaucoma types [16]. However, we were interested in evaluating the outcomes in POAG, a major and leading type of blindness worldwide. Certain variations in the IOP reduction and success rates might exist among different glaucoma entities. Giannaccare et al. reported a 37.8% IOP reduction in eyes with angle-closure glaucoma compared with 20.0% in eyes with POAG and 26.2% in eyes with neovascular glaucoma (NVG) after 6 months [15]. Meanwhile, Hu et al. reported higher IOP reduction and success rates in eyes with primary angle-closure glaucoma (PACG) (36.1% reduction and 80.0% success) than in eyes with POAG (17.7% reduction and 55.6% success), NVG (18.6% reduction and 29.2% success), and traumatic glaucoma (21.6% reduction and 50.0% success) after 3 months [18]. Our 3rd- and 6th-month IOP reduction and success rates are higher than those in both of these studies. Nevertheless, both studies included eyes with refractory glaucoma, and such differences in the IOP reduction and success rates might be attributed to the differences in the ultrasound exposure, number of treated sectors, and baseline IOP. On the contrary, Torky et al. reported a 100% success rate of UCP as a primary intervention for both PACG (10 eyes) and POAG (13 eyes) after 12 months in a group subjected to the same treatment conditions; this rate was lower than that for NVG and uveitic glaucoma, suggesting that secondary glaucoma would have less IOP control [13]. The reduction in aqueous production in secondary glaucoma seems insufficient to compensate for the impaired trabecular drainage pathway. However, the outcomes of UCP seem to be comparable with those of interventions for heterogeneous groups of glaucoma entities. Moreover, the differences in success rates between eyes with POAG and our study is attributed to the big difference in sample size. In their study that included 52 eyes, Denis et al. reported comparable 12-month IOP reduction and success rates of the entire group (36.0% reduction and 48.0% success) compared with those for eyes with POAG (33.9% reduction and 45.0% success) at a 6-s exposure time [12]. Rouland et al. compared the IOP reduction rate between their overall study population and POAG groups, including patients with eyes with refractory glaucoma; they showed 31.0% and 29.0% 12-month IOP reduction rates and a 33.0% 24-month IOP reduction rate in both groups [19]. The IOP reduction rate in our study is comparable with that in previous studies; nevertheless, both studies maintained the same protocol of antiglaucoma medications, and there was no significant change in the burden of medications. The IOP reduction rate in our study is also comparable with that in other studies that included patients with POAG as the majority of their study population. In their study that included 26 patients with POAG, Aptel et al. reported a 30% IOP reduction at 12 months, where the IOP decreased from 28.8 to 19.6 mm Hg, while the number of antiglaucoma medications decreased from 3.6 to 3.1 [11]. Sarmento et al. reported a 12-month IOP reduction rate of 45.6% and an overall success rate of 100% in 14 eyes, where UCP was repeated for better IOP control [20]. Indeed, the IOP control and success rates vary among different studies, which is understandable owing to the differences in the study protocols, success criteria, glaucoma types included, baseline IOP and maintenance of antiglaucoma medications, and acceptance of repeat UCP as an enhancement rather than a failure of the initial UCP procedure. However, UCP seems to yield favourable outcomes for POAG. Notably, eyes with higher baseline IOP and antiglaucoma medication burdens are more prone to failure than their counterparts. Indeed, most previous studies have shown a high IOP reduction rate, reaching 30–40%, while almost maintaining the same antiglaucoma medications. However, such reductions might not be sufficient to reduce the IOP to a level deemed safe to prevent further glaucomatous progression and disc damage in the case of a high baseline IOP, even while maintaining antiglaucoma medications. It is not clearly known if subjecting more ciliary epithelium to ultrasound exposure would result in better IOP control. Hu et al. reported an initial better IOP reduction when exposing the ciliary epithelium to eight sectors of ultrasound than to six sectors; however, such an effect disappeared immediately thereafter and became insignificant [18]. Exposing more ciliary epithelium to coagulative necrosis would further decrease aqueous production, but such an effect is temporary, and the remaining epithelium would compensate for such production. Interestingly, most failures occurred after 6 months because of the high IOP. An explanation is that UCP might yield better initial coagulation of the ciliary body, while inflammatory mediator release would stimulate the uveoscleral pathway; therefore, UCP better reduced the IOP and burden of medications. Nevertheless, re-epithelisation and gradual narrowing of the stimulated uveoscleral pathway will contribute to failure in the future [10,11,21,22].

The most common postoperative complications in our study were cataract development or progression, prolonged or rebound anterior chamber reaction, hypotony with choroidal detachment, and macular oedema. All these complications were non-vision-threatening complications and shared a common predisposing factor, which is the release of inflammatory mediators after the disruption of the blood–aqueous barrier induced by ciliary epithelium coagulative necrosis. Such inflammatory mediators will spread to the anterior chamber and result in inflammation and cataract development or progression, stimulate the uveoscleral pathway and result in choroidal detachment with hypotony, or spread to the vitreous humour and result in the development or aggravation of macular oedema [16,17].

## 5. Conclusions

Although the current study had the limitations inherent in any retrospective study, it is the first to evaluate the outcomes of UCP for a major glaucoma entity, such as POAG. A significant IOP control and reduction of the antiglaucoma medication burden can be achieved with UCP. However, such effects could be less in cases of higher baseline IOP and antiglaucoma medication burden. Therefore, counselling patients with such conditions regarding the need for enhancement or other surgical modalities is recommended.

## Figures and Tables

**Table 1:** Characteristics and ocular history *.

Variable	(N = 66)
Age at the time of surgery, year **	60.77 (±12.8)
Sex **	
Male	31 (53.0%)
Female	24 (47.0%)
Previous glaucoma surgery	
Yes	29 (43.9%)
No	37 (56.1%)
Frequency of glaucoma surgery	
1	27 (40.9%)
2	2 (3.0%)
Type of previous glaucoma surgery ǂ	
Trabeculectomy + MMC ± Phaco + PCIOL	11
Deep sclerectomy + MMC ± Phaco + PCIOL	11
Cyclophotocoagulation ± Phaco + PCIOL	5
Ahmed implant	2
Canaloplasty + MMC ± Phaco + PCIOL	1
Previous non-glaucoma surgery	
Yes	31 (47.0%)
No	35 (53.0%)
Type of previous non-glaucoma surgery ǂ	
Phaco + PCIOL	28
Pars plana vitrectomy	3
Lens status	
Phakic	26 (39.4%)
Pseudophakic	39 (59.1%)
Aphakic	1 (1.5%)
Axial length, mm	24.50 (±2.5)
White-to-white, mm	11.84 (±0.5)
Cone size used	
11	3 (4.5%)
12	32 (48.5%)
13	31 (47.0%)

* Data are presented as means (±SDs) and frequencies (%). Numbers are per eye. ** Numbers are per patient. ǂ Number represents the frequency of overall surgeries, including repeat surgeries. MMC: Mitomycin C. Phaco: Phacoemulsification. PCIOL: Posterior chamber intraocular lens implantation.

**Table 2:** IOP, number of antiglaucoma medications, and logMAR.

Preoperative Baseline		*p*-Value
IOP, mm Hg	23.02 (±6.1)	-
No. of medications	3.23 (±0.9)	-
LogMAR	0.67 (±0.7)	-
No. of eyes	-	-
1 day postoperative		
IOP, mm Hg	15.10 (±6.1)	<0.01
IOP reduction (%)	34.82%	-
No. of medications	1.25 (±1.3)	<0.01
LogMAR	0.73 (±0.8)	0.02
No. of eyes	66	-
1 month postoperative		
IOP, mm Hg	16.54 (±5.5)	<0.01
IOP reduction (%)	28.84%	-
No. of medications	1.67 (±1.4)	<0.01
LogMAR	0.83 (±0.9)	0.12
No. of eyes	66	-
3 months postoperative		
IOP, mm Hg	17.20 (±6.7)	<0.01
IOP reduction (%)	32.36%	-
No. of medications	1.82 (±1.4)	<0.01
LogMAR	0.90 (±1.0)	0.03
No. of eyes	64	-
6 months postoperative		
IOP, mm Hg	17.04 (±5.5)	<0.01
IOP reduction (%)	41.07%	-
No. of medications	1.83 (±1.5)	<0.01
LogMAR	0.78 (±0.9)	0.16
No. of eyes	64	-
9 months postoperative		
IOP, mm Hg	15.60 (±4.8)	<0.01
IOP reduction (%)	42.03%	-
No. of medications	1.90 (±1.5)	<0.01
LogMAR	0.78 (±0.9)	0.18
No. of eyes	58	-
12 months postoperative		
IOP, mm Hg	18.22 (±7.0)	<0.01
IOP reduction (%)	35.51%	-
No. of medications	2.15 (±1.5)	<0.01
LogMAR	0.64 (±0.7)	0.55
No. of eyes	47	-
18 months postoperative		
IOP, mm Hg	16.39 (±4.4)	<0.01
IOP reduction (%)	39.57%	-
No. of medications	2.00 (±1.6)	<0.01
LogMAR	0.63 (±0.9)	0.37
No. of eyes	38	-
24 months postoperative		
IOP, mm Hg	16.44 (±5.3)	<0.01
IOP reduction (%)	38.04%	-
No. of medications	2.09 (±1.6)	<0.01
LogMAR	0.64 (±1.0)	0.7
No. of eyes	27	-

Data are presented as means (±SDs). P-values are calculated using the Wilcoxon test and Student’s *t*-test. IOP: Intraoculr pressure, logMAR: logarithm of minimal angle of resolution.

**Table 3:** Postoperative complications following ultrasound cyclo plasty *.

Complication	Number (%)
Cataract development or progression ǂ	8 (30.8)
Prolonged or rebound anterior chamber reaction	7 (10.6)
Hypotony or choroidal detachment	2 (3.0)
Macular oedema	2 (3.0)
IOP spike of >30 mm Hg	1 (1.5)
Corneal abrasion	1 (1.5)

* Numbers are per eye. ǂ Percentage out of phakic eyes.

**Table 4:** Cox proportional hazard regression analysis.

Predictor	HR	95% CI	*p*-Value
Age	0.99	0.97–1.01	0.62
Sex	1.16	0.69–1.97	0.58
Baseline IOP	1.10	1.00–1.12	0.04
Baseline no. of medications	3.01	1.61–5.60	<0.01
Old glaucoma surgery	1.18	0.70–1.98	0.54
Old non-glaucoma surgery	0.64	0.37–1.10	0.11
Axial length	0.99	0.85–1.15	0.92
White-to-white-diameter	0.78	0.40–1.50	0.78

HR: Hazard ratio; CI: Confidence interval; IOP: Intraocular pressure.

**Table 5:** Eyes needing repeat glaucoma surgery.

First Repeat Surgery	Time Since the First Procedure (Month)	Second Repeat Surgery	Time Since the First Repeat Procedure (Month)
Ahmed implant	19.3		
Phaco + PCIOL * + ECP **	8.8	Micropulse cyclophotocoagulation	12
Ahmed implant	19.3		
Deep sclerectomy + MMC ǂ	11.5		
Trabeculectomy + MMC	7.8		
Ahmed implant	19.3		
Deep sclerectomy + MMC	12.7		
Deep sclerectomy + MMC	12.2	Ahmed implant	24
Phaco + PCIOL + trabeculectomy + MMC	6.2	Trabeculectomy + MMC	6
Micropulse cyclophotocoagulation	8.8		
Express shunt + MMC	12.3		
Express shunt + MMC	10.1		
Cyclophotocoagulation	23.9		

* Phacoemulsification with posterior chamber intraocular lens implantation; ** Endoscopic cyclophotocoagulation; ǂ Mitomycin C.

## Data Availability

All data are available from the corresponding author upon request.
